# Pathways to potentially preventable hospitalizations for diabetes and heart failure: a qualitative analysis of patient perspectives

**DOI:** 10.1186/s12913-016-1511-6

**Published:** 2016-07-26

**Authors:** Tetine L. Sentell, Todd B. Seto, Malia M. Young, May Vawer, Michelle L. Quensell, Kathryn L. Braun, Deborah A. Taira

**Affiliations:** 1 Office of Public Health Studies, University of Hawai‘i at Manoa, 1960 East-West Road, Biomed, Honolulu, HI 96821 USA; 2 Queens Medical Center, 1301 Punchbowl Street, Honolulu, HI 96813 USA; 3 1301 Punchbowl Street, Honolulu, HI 96813 USA; 4 Daniel K. Inouye College of Pharmacy, University of Hawai‘i at Hilo, 677 Ala Moana Boulevard, Suite 1025, Honolulu, HI 96813 USA

**Keywords:** Hospitalization, Diabetes, Heart Failure, Asians, Pacific Islanders, Readmissions

## Abstract

**Background:**

Potentially preventable hospitalizations (PPH) for heart failure (HF) and diabetes mellitus (DM) cost the United States over $14 billion annually. Studies about PPH typically lack patient perspectives, especially across diverse racial/ethnic groups with known PPH health disparities.

**Methods:**

English-speaking individuals with a HF or DM-related PPH (*n* = 90) at the largest hospital in Hawai‘i completed an in-person interview, including open-ended questions on precipitating factors to their PPH. Using the framework approach, two independent coders identified patient-reported factors and pathways to their PPH.

**Results:**

Seventy-two percent of respondents were under 65 years, 30 % were female, 90 % had health insurance, and 66 % had previously been hospitalized for the same problem. Patients’ stories identified immediate, precipitating, and underlying reasons for the admission. Underlying background factors were critical to understanding why patients had the acute problems necessitating their hospitalizations. Six, non-exclusive, underlying factors included: extreme social vulnerability (e.g., homeless, poverty, no social support, reported by 54 % of respondents); health system interaction issues (e.g., poor communication with providers, 44 %); limited health-related knowledge (42 %); behavioral health issues (e.g., substance abuse, mental illness, 36 %); denial of illness (27 %); and practical problems (e.g., too busy, 6 %). From these findings, we developed a model to understand an individual’s pathways to a PPH through immediate, precipitating, and underlying factors, which could help identify potential intervention foci. We demonstrate the model’s utility using five examples.

**Conclusions:**

In a young, predominately insured population, factors well outside the traditional purview of the hospital, or even clinical medicine, critically influenced many PPH. Patient perspectives were vital to understanding this issue. Innovative partnerships and policies should address these issues, including linkages to social services and behavioral health.

**Electronic supplementary material:**

The online version of this article (doi:10.1186/s12913-016-1511-6) contains supplementary material, which is available to authorized users.

## Background

Hospitalizations for heart failure (HF) and diabetes mellitus (DM) cost the United States over $14 billion annually [1], yet many of these hospitalizations are considered “potentially preventable hospitalizations” (PPH). PPH are believed to be avoidable with better access to high quality outpatient care [2]. Preventable readmissions are a particular focus of current health policy, with penalties for hospitalizations that have high readmission rates [3].

Identifying reasons for PPH and readmissions are critical, particularly to help inform system and organizational-level interventions [4, 5]. Gaining insight into socio-demographic factors is particularly important, as a handful of previous studies have found that social factors impact the risk of hospital readmission, though research on this topic is still limited [6–11]. The vast majority of studies about these topics utilize administrative data, which typically lack key socio-demographic variables as well as patient perspectives about their PPH [6].

Patients can provide key insight into their reasons for hospitalization and their specific challenges in outpatient management, which are critical to designing effective interventions [8–11]. Patient perspectives are also vital to the mission and success of the growing field of patient-centered outcomes research (PCOR), which requires “that the patient’s voice and perspective drive every step of the research process, including prioritizing the research questions, designing and conducting the research, and implementing the results in practice” ([12], pg. 6). Including patient voices is particularly important across diverse racial/ethnic groups with distinct cultural perspectives and health correlates [13].

The study goal was to understand patient perspectives of factors that led to a PPH for heart failure and diabetes so that these insights could be considered in research, clinical practice, and policy. We focused on these two conditions because they include the most common preventable hospitalizations, responsible for 39 % of all PPH [1]. This study presents the in-depth qualitative results from a larger mixed-methods study focusing on identifying and understanding disparities in PPH for heart failure and diabetes across diverse racial/ethnic groups, particularly Asian American and Pacific Islander populations. Better understanding predictors of PPH in these populations has urgency, as disparities have been found in PPH rates and outcomes for some Asian American and Pacific Islander population subgroups, especially Native Hawaiians [14, 15].

Specifically, the qualitative findings presented here illuminate patient-identified factors and narratives of why they felt they were hospitalized with a PPH. From these findings, we developed a model to understand an individual’s pathways to a PPH through groupings of factors that we term *immediate* (urgent clinical reason for the admission), *precipitating* (practical explanation for that urgent clinical reason), and *underlying* factors (fundamental drivers of the practical challenges), which could help identify potential intervention foci. We also demonstrate our model’s utility using five examples from our study sample.

## Methods

### Study population

All subjects were recruited from The Queen’s Medical Center (QMC), which is the largest hospital in Hawaii and the primary tertiary medical referral center for the Pacific Basin [16]. From June 2013-December 2014, adults hospitalized at QMC for HF or DM-related PPH were identified for study admission using Agency for Health Care Research and Quality (AHRQ) metrics [2]. Specifically, eligible subjects had a primary admission diagnosis of at least one of the following four conditions, as defined by the AHRQ definition of PPH: uncontrolled diabetes, short-term diabetes complications (e.g., ketoacidosis, hyperosmolarity) and long-term diabetes complications (e.g., renal, eye, circulatory), and lower-extremity diabetes-related amputations. Reflecting the predominant racial/ethnic populations of Hawaii, we restricted recruitment to participants categorized as Asians, Native Hawaiians, other Pacific Islanders (e.g., Samoans, Tongans, Micronesians), and whites. Participants also needed to be proficient in English.

### Recruitment

Potentially eligible subjects (across the basic three domains: condition, race/ethnicity, and English language proficiency) were identified in one of two ways. First, the patients’ attending physician could identify and refer potentially eligible subjects to the research team. Attending physicians for these patients were typically cardiologists (for heart failure), hospitalists or other internists (for DM complications and heart failure), or vascular surgeons (for lower-extremity amputations). Second, advance practice nurses (APRNs) who were part of the care team identified potentially eligible subjects, and notified the research team. Prior to contacting the patient to obtain informed consent, the patient’s attending physician was contacted for permission to approach the patient. During the recruitment period, the research nurse communicated with the APRNs and/or other providers approximately two times a week to discuss the study and identify patients who met inclusion criteria.

Once the attending physician’s permission was obtained, a trained research nurse approached each potential study subject to confirm study eligibility based on face-to-face interview. Once study eligibility was confirmed, all interested subjects provided written informed consent for the study, which included a 40-min interview and a review of relevant data from patients’ electronic medical records.

Patients were excluded who were: 1) Age <21 years old; 2) Unwilling to participate in semi-structured interview and answer survey questions; 3) Did not self-identify as Asian, Native Hawaiian, other Pacific Islander, or white; 4) In the Intensive Care Unit; 5) Clinically unstable; 6) Pregnant; 7) With memory loss or unable to participate in interview; 8) Non-Hawaii resident; or 9) A resident of nursing home, hospice, prison, or other similar institution.

Sampling was stopped when thematic saturation on patient-reported factors was reached. Due to the diverse nature of the study population (especially in terms of age, race/ethnicity) as well as the different types of hospitalizations examined, our study population was large to ensure all relevant themes were identified.

### Sample

A considerable portion of individuals identified as potentially eligible by providers (*n* = 393) were ineligible for the study interview (*n* = 238). Top reasons were altered mental status, including dementia (*n* = 82), limited English proficiency (*n* = 76), and being too ill to participate (*n* = 34). Among those otherwise eligible, 30 refused participation, mostly stating they were not interested in the topic. An additional two individuals were deemed ineligible for study inclusion after interview completion; one admission was for a congenital heart issue, not a preventable condition, and one was for chronic obstructive pulmonary disease, not a study focus. Thus, the final interview sample was 90. Participants received a $20 drug store gift card for participation.

### Questionnaire

Following consent, participants completed the Rapid Estimate of Adult Literacy in Medicine [17, 18], which took 3–5 min, followed by a semi-structured oral questionnaire administered by the interviewer. (Relevant items used from the questionnaire to provide useful background data for the qualitative analyses are included as Additional file 1). The questionnaire took approximately 20 minutes and was given first to provide time to develop the relationship with the participant, including establishing listening, rapport, and trust, that would create the foundational context for next portion of the interview, the open-ended questions. Questionnaire responses were collected using REDCap [19].

### Open-ended questions

The open-ended questions followed the oral questionnaire. Examples are attached in Table 1 and all open-ended questions are included in Additional file 1. These questions were designed to elicit patient perspectives on their reasons for hospitalization for the PPH. Probes were used to encourage patients to elaborate on stories and themes when needed. Answers to open-ended questions were recoded using a tablet computer. The open-ended questioning took approximately 20 min.

**Table 1:** Sample open-ended questions

Give me a sense of what was going on at home and with your health before you came to the hospital.	
Is there anything different that could have been done to prevent you from coming to the hospital? Anything your doctor could have done?	
Are there any things you will do differently when you go home from the hospital this time?	

### Field notes

The interviewer also compiled field notes on the study questionnaire, particularly when patients added more detail following from one of the structured interview questions. These notes were also used to understand patients’ stories and pathways.

### Medical record review

Additional data were obtained through medical record review, including baseline demographic information (e.g., age, gender) and relevant clinical information (e.g., same hospital readmission). Relevant items from the study instrument used for medical record review that were used to contextualize the study sample for the qualitative analysis is attached as Additional file 2.

### Descriptive analyses

STATA 12.0 (College Station, 2011) was used for descriptive analyses of relevant demographic and clinical data from the interview.

### Qualitative analyses

Two independent coders (MQ, TS) with expertise in chronic disease, social factors in health, and/or Asian and Pacific Islander communities considered interview transcripts (for the first 20 patients), audio recordings (for all participants), and interview field notes (for all participants). Both coders first reviewed and coded all material independently. This included listening separately to the recordings of the open-ended responses. We chose to listen to the audio-recordings for coding after comparing the transcripts for the first 20 respondents with the audio recording for those patient interviews and noting important emotional details that were not present in the transcripts. Listening to the recordings was time consuming, but essential to fully understand patients and their stories of their pathways to hospitalization.

Coders meet at least biweekly and used an iterative approach to confirm themes and pathways. Patients were reviewed in batches of approximately 10–15 at a time. Coders first identified themes and considered each interview separately according to these themes. As coders met to review and discuss findings, they revised the template of themes and proposed patient pathways. Coders reviewed audio-recording together as needed to reach consensus on each participant. We also meet regularly with the study team (which included a provider, a researcher, and a qualitative experts) to discuss and contextualize emerging results. After all 90 patients were reviewed, coders re-reviewed consensus coding documents to ensure their congruence with the final study factors and pathways. The final consensus coding was used for analyses [20].

Analyses were guided by the “framework approach” [21], a qualitative method that merges the case and themes approaches. Coders considered patient-reported reasons for their preventable hospitalizations, capturing expected factors from previous research and theory (e.g., transportation, medication adherence, access to care [4, 8, 10]) and emerging factors based on patient stories. As reasons for these hospitalizations were multidimensional [8], one individual’s hospitalization story might include multiple reasons for the same PPH. Some of the expected factors based on previous research and theory included challenges in transportation to care, medication adherence, access to care, and comprehension of relevant medical information. Some of the themes that emerged were patients feeling of denial/avoidance of the problem leading to their hospitalization as well as substance abuse as a precipitating factor. The other emerging finding was the overall framework (our study model described in more detail below) in which the stories patients told of their reasons for hospitalization fell into layered immediate, precipitating, and underlying factors. A completed COREQ checklist with more detail regarding the qualitative analyses study methods is attached as Additional file 3.

## Results

Table 2 is included to provide context on the study sample. Of the 90 participants, 72 % were under 65 years (mean age 55.7 years; SD 13.3), and 30 % were female. Interestingly, 90 % of the sample had health insurance, and 88 % had a usual source of care. From the administrative data, 29 % of hospitalizations were HF-related, 34 % DM-related, and 37 % both HF and DM-related. More than half (66 %) of respondents were previously hospitalized for the same clinical condition, many with multiple readmissions, making this a population of high policy relevance given for PPH and potentially preventable readmissions. The study sample was ethnically diverse; 40 % was Native Hawaiian, 16 % was other Pacific Islander, 13 % was Asian, 12 % was Filipino, and 19 % was white. Over 53 % of the study sample had low health literacy as measured by the REALM. Most of the study sample was low income, with over 35 % reporting family incomes of less than $20,000 year and an additional 22 % reporting family incomes below $40,000.

**Table 2:** Descriptive results for study participants overall (*n* = 90)

	Total
Demographics	n (%)
Race/Ethnicity	
Asian	12 (13.3)
Native Hawaiian	36 (40.0)
Other Pacific Islander	14 (15.7)
Filipino	11 (12.2)
White	17 (18.9)
Education	
< High School	20 (20.2)
High School Graduate	66 (73.3)
College Graduate	3 (3.3)
College Degree+	1 (1.1)
REALM	
Low Health Literacy (<12 Grade Reading Level)	48 (53.3)
Age Group	
18–64	64 (71.1)
65+	26 (28.9)
Female	
	30 (33.3)
Family Income	
< $20,000	32 (35.6)
$20,000–$39,999	20 (22.2)
$40,000+	9 (10.0)
Missing	29 (32.2)
Insured	
	81 (90.0)
Usual Source of Care	
	78 (87.6)
Same-Hospital Readmission	
Any Readmission	55 (65.5)
Average number of CVD Readmissions (range 1–6)	1.97 (SD:1.28)
Average number of DM Readmissions (range 1–6)	2.11 (SD: 1.29)
Type of hospitalization (from chart)	
Diabetes	31 (34.4)
Heart Disease	26 (28.9)
Both Diabetes and Heart Disease	33 (36.7)

### Pathways

**Fig. 1:**
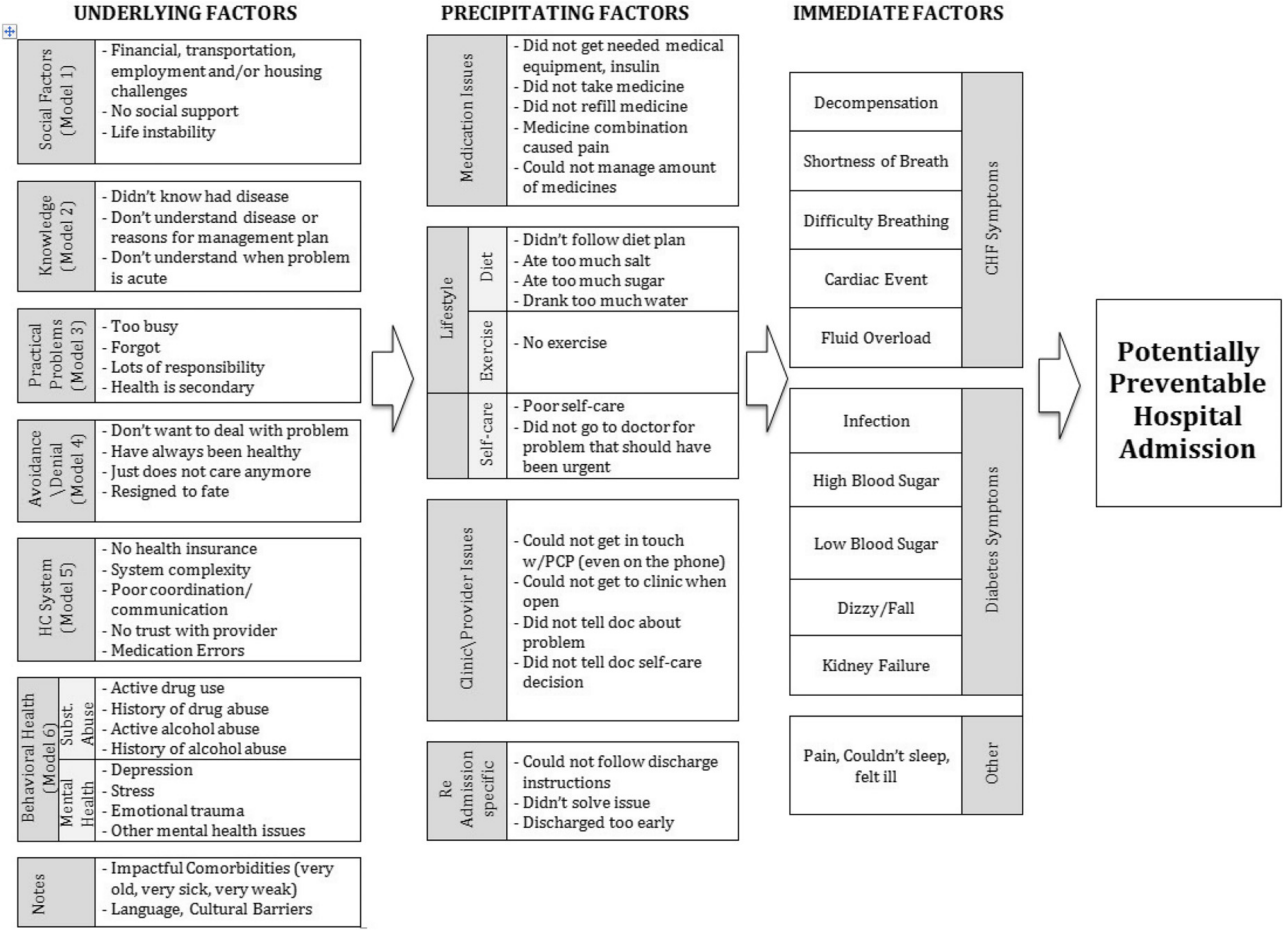
Model of underlying, precipitating, and immediate factors resulting in potentially preventable hospitalizations from patient stories

Many themes expected from existing scholarship (homelessness, financial challenges, access to care) were seen in patient stories. Additional factors emerged from patient stories, including patient “denial/avoidance” of their illness and trust issues with providers. After careful analysis of the patterns of these factors from patient stories, it became clear that patient stories suggested pathways to PPH influenced by distinct sets of reasons, which we term *immediate, precipitating,* and *underlying* reasons (Fig. 1). The *immediate* reason was the urgent reason (e.g., shortness of breath, infection) that caused the individual to be admitted to the hospital. The *precipitating* reason was what specifically happened that led directly to the urgent matter that necessitated the hospital admission (e.g., did not take medication, visit doctor, or follow diet). Finally, patients typically described deeper *underlying* reasons for their PPHs. The *underlying* reasons emerged from patient stories explain why they did not take medication, visit a doctor, or follow their chronic care management plans.

### Immediate reasons

According to patient stories, 47 % initially went to the hospital for an acute issue related to HF (especially shortness of breath/trouble breathing), 46 % went for an acute issue related to DM (especially infection/amputations), 9 % went for an acute issue related to pain, and 15 % went because they felt very ill. Because patients could report multiple reasons, the sum exceeds 100 %, and patient-reported reasons for admission did not necessarily match the coded hospitalization-type from administrative data.

### Precipitating reasons

Patients identified four types of challenges precipitating their PPH. These challenges were related to medication (57 %), self-care/lifestyle (47 %), the clinical encounter (44 %), and a recent, previous hospitalization with inadequate resolution (24 %). Medication-related challenges included patients not refilling their medications, not taking medications at the right time or dose (or at all), or not obtaining necessary medical equipment (e.g., needles) to take medication. The biggest lifestyle and self-care challenge was following the prescribed diet. A common clinic-encounter-related challenge was difficulty getting to the clinic or contacting the clinician, especially on the weekend or holidays. Under readmission-related challenges, several patients felt they were discharged too early or received inadequate care during the previous hospitalization. Others gave reasons why they could not follow critical discharge instructions (e.g., one stopped taking prescribed medication because he already felt better; one was discharged with instructions not to walk, but went home and had to do household chores that required walking and standing).

### Underlying reasons

From the patients’ perspective, challenges in chronic care management and interactions with the health system that led to their precipitating condition were associated with the social, emotional, clinical, and financial context of their lives. These were grouped into six, non-exclusive sets of underlying factors: extreme social vulnerability, health system interaction issues, limited knowledge, behavioral health issues, denial of illness, and practical problems. Most individuals reported multiple underlying factors impacting their chronic care management and/or access to care. While the critical importance of social and behavioral factors to health is well known [22], these are not well described in relationship to PPH. Thus, we focus in detail on findings related to *underlying* factors.

#### Social vulnerability

The most common underlying factor was *social vulnerability*, reported by 54 % of respondents. This was supported by the demographic data; only 5 % of patients interviewed had at least a college degree, 53 % had a family income under $20,000/year, and 53 % had low health literacy. Significant underlying social vulnerabilities, including housing insecurity, limited social support, lack of transportation, employment or financial issues, or, most often, a combination of these factors, led to problems in chronic care management, which, according to patients’ stories, eventually left them so ill they had to be hospitalized.

A 76-year-old woman hospitalized with diabetes and heart disease stated: “*Basically it’s a day-to-day struggle. It’s stressful because the cost of keeping healthy is expensive and I have no money.*” Another 53-year-old female with both diabetes and heart disease noted, “*I am not lazy…[It] costs a lot of money for me to just do that [eat healthy]. [It’s] so expensive to make my own soup.*”

In a specific example, when a recently discharged 65-year-old man with both heart disease and diabetes went back to the place he had shared with a number of roommates, the electricity was turned off. He said: *“I…couldn’t eat what I wanted to eat… I felt panicked because I cannot do nothing.*” He also had no consistent social support. His daughter was in jail, and he relied on his daughter’s girlfriend for transportation.

In many cases, individuals who described these extreme social vulnerabilities noted that they were aware of what they should be doing to manage their chronic condition, but were unable to implement the plan because of their social or financial circumstances. For instance, a 50-year-old woman who said, “*Because we live on street, it’s hard to get clean, running water. It’s hard to find time to take the meds, especially if you have to do it three times a day with meals*.”

#### Health care system

*Health care system* interaction issues, reported by 44 % of respondents, included two general themes. One was an issue of insufficient interaction with the health care system due to lack of insurance or access. A 33-year-old male with heart disease stated: “*I’m not taking nothing [medicine] for pretty long and didn’t go see anyone for help because I didn’t have medical insurance. I wasn’t working and didn’t have medical so I didn’t honestly really think I could actually come in. And yes, I didn’t have any money to pay for my medicine.*” A common link between patient-reported social vulnerability and health care insurance/access is also seen in this example.

However, as most respondents had insurance (90 %) and a usual source of care (88 %), the other theme of challenges in health care communication/coordination was more common. This included stories of providers (in primary care, the hospital, and specialists) not taking time with the patient, using overly complex words, not having local and/or cultural knowledge as well as issues of trust. Patients also reported feeling like providers did not respect them and noted communication issues within the health care system—like doctors not taking to pharmacists, and specialists not talking to primary care. Many patients had multiple doctors and health conditions, making communication across providers and/or pharmacies important.

A 39-year-old woman with diabetes said: “*They do it so fast, you say yah, yah, because they in one rush and you know they’ve got to do something else. They got no more the time for talk with you or sit and find out cause they get other patients they got to go make money off of*.”

The sense of lack of respect and trust was particularly notable among those with substance abuse histories. A 48-year-old female with diabetes and methicillin-resistant Staphylococcus aureus (MRSA) who was an active methamphetamine user with many previously hospitalizations and amputations said:

*“I trust only certain doctors, not all…To be honest, I don’t trust the in-house doctors because they are not listening to me… I just feel like I am just a guinea pig for them….This morning I had 4 of them standing in front of me, poking at me, picking at me….It took them almost 5 days to get me pain medicine. They thought Tylenol was going to do it…It’s not for pain when you have MRSA. It’s not going to work, I told them. But they didn’t listen to me until my infectious doctor came in and ordered something for the pain. That was 5 days later.”*

While many patients noted these communication issues with their doctors, due to cultural norms, few were likely to mention these challenges to their doctors. A 73-year-old woman with both diabetes and heart disease stated: *“Sometimes I don’t understand the doctor, and I don’t do what I’m told. I never think about asking him to repeat. It’s part of my culture not to ask or question. Because he is not from my culture and sometimes he does not understand my needs.”*

There was also an overlap with language, as exemplified by this 23-year-old female respondent with diabetes: “*[It’s] easier for me to understand in [my language]. I never ask for an interpreter, nor has anyone asked if I needed one. I want to do the right thing, but [I am] scared and shamed and I don’t know how to ask. I don’t understand why, when, what to do. I get scared and feel overwhelmed.*”

#### Knowledge

Often related to communication/coordination challenges noted above, 42 % of respondents reported *lack of knowledge*. A few individuals simply did not know that they had a condition to manage and thus became dangerously ill. However, most had the disease for many years. Many had been previously hospitalized for an identical (or related) issue. Still, they lacked specific information about how to care for their condition, a deep understanding of their condition, and/or how to identify warning signs related to the condition. Many individuals echoed the thoughts of this respondent, a 52-year-old man with diabetes: “*I didn’t know how serious my condition was. I was shocked to see how much of my toe was removed.*”

Another participant, a 80-year-old woman with heart disease, was eating a lot of hidden salt because, though she knew general chronic care management information (e.g., “eat no salt”), she didn’t know how this applied to the specifics of her day-to-day life (e.g., how much salt is in the foods she actually eats) or the consequences. She said: “*Talking to docs was just fine but…didn’t think a little salt would hurt.*” In another case, a 66-year-old man with both diabetes and heart disease knew about his disease, but believed he did not need to monitor his insulin when he was taking his medication: “*I don’t think I need to poke my finger. Only when you come here [hospital] I guess your body gets stressed so you gotta do that. But when I go back home…my pills goin’ cover.*”

Another common issue was the patient considering insulin/needles and/or Lasix as distinct from “medication.” For instance, an 80-year-old woman with heart diseases said she sometimes she forgets her “*water pills,*” but would never forget her “*medication.*”

#### Behavioral health

*Behavioral health issues* were reported by 40 % of respondents. Among these, 37 % reported substance abuse, typically methamphetamines or alcohol, 37 % reported mental health issues, most commonly depression, and 27 % had both active substance abuse and mental health issues.

This factor was only coded when respondents specifically reported mental health and/or substance abuse in their own stories of their hospitalization. Other individuals had behavioral health issues noted in their chart, but did not include these factors in their narratives of chronic care management, self-care, or motivational challenges that led to their hospitalization. Thus, the role of behavioral health issues is likely underestimated, either because some patients were unwilling to disclose substance abuse or mental health issues and/or did not feel these issues played a significant role in their PPH story.

Many respondents did specifically report these background behavioral health factors as being the reason for their lack of chronic care management that led to their hospitalization. As described by a 41-year-old male with heart disease: “*I lost my wife, and then I couldn’t focus at work, so I lost my job. I lost my house and I did drugs cause I didn’t want to live no more. I just wen’ stop taking my medicines*.”

Many did not tell their doctors about their substance use or depression, feeling like the following respondent, a 25-year-old female with both diabetes and heart disease, “*I don’t like talking about depression or sadness. I tell my mom…but don’t tell doctor.*” Others were like a 33-year-old male with heart disease who, when probed, mentioned that on the weekends he drinks 24 beers and 2 bottles of Jack Daniels. He said his cardiologists did not know about his about drinking “*because he never asked me.*”

Reported substance use factors were often associated with poor interactions with the health care system as described by a 68-year old man with diabetes, previous amputations, and extensive alcohol abuse. He felt he was hospitalized because, though he had lots of doctors, they “don’t know sh** about anything,” and **they** gave him the “*run around*.”

#### Denial/Avoidance

*Denial or Avoidance* of the problem was reported by 27 % of respondents. Individuals reported knowing how to better manage their chronic health condition, but specifically stated that they were in denial about their problem and had not wanted to address such a stressful and demanding issue that would necessitate significant changes in their lifestyle, quality of life, or sense of self.

A 52-year-old male with both diabetes and heart disease said: “*Took me a long time cause I’m real stubborn…I refused to admit what I have. I refused to admit I’m diabetic.*” Another participant, a 53-year-old female with heart disease and diabetes was not interested in receiving patient education: “*I am not going talk to a dietician cause I going do and eat what I want anyway. I’m stubborn.*”

Several patients described how the clinical diagnosis challenged their self-perception. A 74-year-old man with diabetes and heart disease said, “*It’s hard to accept myself because I cannot function like I used to*.” Before this illness, he was a strong man with a very physical job delivering appliances. He explained that because he doesn’t fully embrace his diagnosis, he didn’t change his diet. As with many individuals describing this pathway, he said this hospitalization was a wake-up call to take his disease more seriously: “*I tried to live [my] own way. It didn’t work.*”

Others reported that they avoid chronic care management not so much due to depression (though these could be linked), but due to a fatalistic attitude. A 59-year-old man with both diabetes and heart disease said: “*I cannot say I forget [to take meds]- just lazy. I feel like it’s a waste of time…The way I look at it- different than you medical [people] think about. I know that we all gonna die someday. How, when, we don’t know. So I already have accept …. I just want to enjoy what I have left.*”

#### Practical problems

*Practical problems* not due to social or behavioral health circumstances or denial/avoidance, but just due to logistics, such as being too busy to manage the condition, were the final set of *underlying* factors, reported by 6 % of respondents. A 65-year-old man re-hospitalized with diabetes said he had “*no time for sitting around and learning math to take care of myself and having to cope with my disease…I had to feed chickens and cows.*” A 38-year-old mother with diabetes noted: “*They just tell me the normal things. You’ve got to take your meds, your insulin…but …to be at home taking kids and to do all of that ain’t so easy.*”

### Examples of pathways

**Fig. 2:**
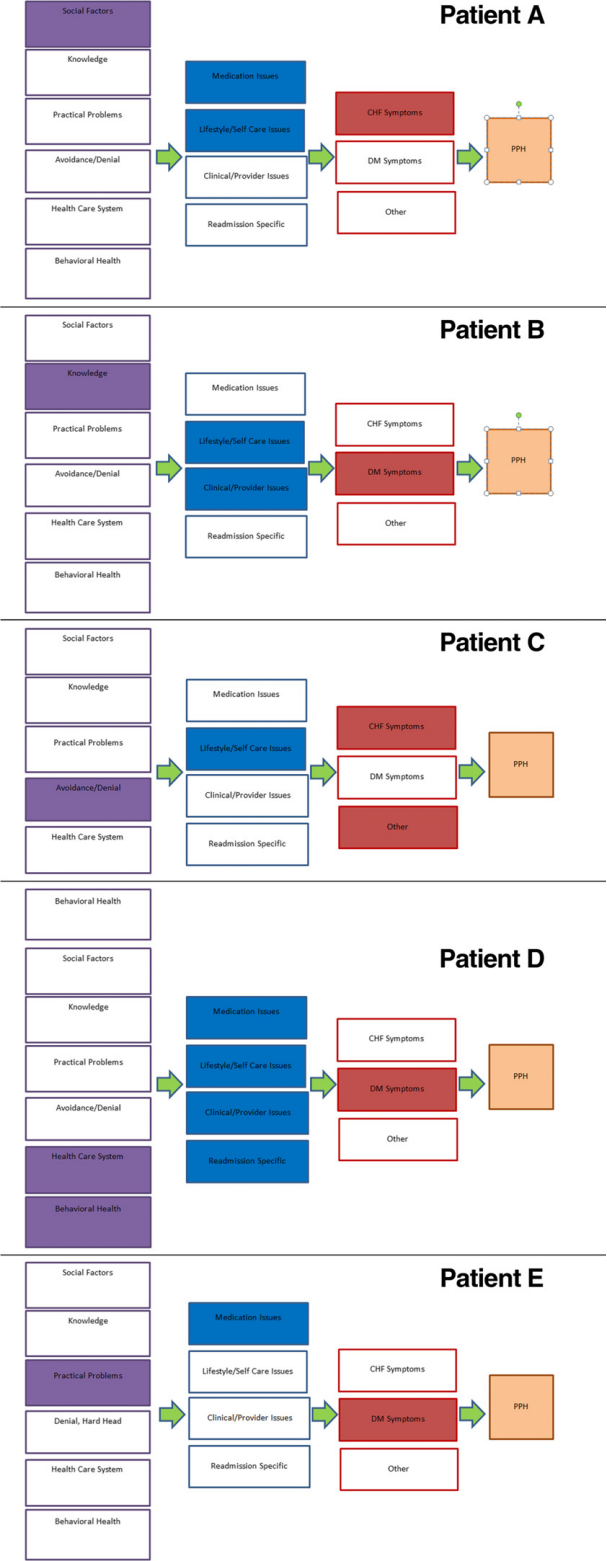
Pathways from patient stories. Each of the three pathway steps has a color for orientation. When relevant, the factor within this pathway is chosen by filling it in. This process could allow for gradation in the strength of the factor as factors of critical importance could be highlighted darkly while factors present, but of lesser importance in explaining the potentially preventable hospitalization, could be highlighted at a lower color value. **Patient A:** 52-year-old male with congestive heart failure. **Patient B:** 62 year-old female with diabetes. **Patient C:** 57-year-old male with congestive heart failure. **Patient D:** 39-year-old female with diabetes. **Patient E:** 41-year-old male with diabetes

Drawing from patient stories of *underlying* reasons for the admission described in detail above, along with *immediate* and *precipitating* factors*,* the model seen in Fig. 1 can be used to determine pathways for preventable hospitalizations and thus can be used to direct interventions. We consider the utility of the pathways in understanding the problem and in directing interventions using five examples drawn from the study sample that highlight use of the model in clinical care (Fig. 2).

#### Example 1: Social factors

*“I look at my medications, says: take with food, don’t take on an empty stomach…I never have money. I never have food. I couldn’t buy me food…Sometimes I got to the churches, in between, in the middle of the month when I run out of food stamps and I run out of money, social security money. I got to the churches, but a lot of the food is canned good stuff… It contributes a whole lot, and not eating healthy and poor diet.”*

Patient A is a 52-year-old male with heart failure. He was hospitalized because he could not breathe, which was his *immediate* reason for hospitalization. This was a re-admission; he had been hospitalized the week before. The *precipitating* reasons for this hospitalization were noncompliance with prescribed medication and diet. The *underlying* factor, however, was extreme social vulnerability due to low income, homelessness, and limited social support, as his daughter lives in another community. He has a car, which he sleeps in, but no money for gas and no phone. He requested help obtaining a disability bus pass during the study interview.

Reducing or eliminating PPH for Patient A may involve addressing his housing, transportation, social support, and financial challenges or he will likely struggle with the same chronic care management issues, despite even the most excellent patient education, health care access, or case management.

#### Example 2: Knowledge

*“What I was thinking about my toe? Nothing. I was just surprised when my toe looked like that. My diabetes never show out, never show out that I have that problem…There was not sign for diabetes on myself.”*

Patient B is a 62-year-old female with diabetes covered by Medicaid. Her *immediate* reason for hospitalization was gangrene/cellulitis, and her toe was subsequently amputated. She also has diabetic retinopathy. The *precipitating* reason for Patient B’s hospitalization was that she did not see a doctor when her toe became infected. The *underlying* reason for this was a lack of knowledge.

But, as was the case for most participants, the issue was not a complete lack of knowledge. Patient B was monitoring her foot during this time. She knew why she should do this and was checked regularly by her doctor. As she stated: *“Diabetes when you already have that sickness, you cannot feel if you step on something hard so every time I visit the doctor he touch, ‘you feel this, you feel this,’ on my toes.”* Despite this general awareness and monitoring, she described that she did not fully understand what she was looking for and did not know when a problem was acute. Rather, her daughter convinced her to see a doctor.

Like many participants, Patient B had significant gaps in her knowledge about her illness that limited her self-care, despite the fact that she had diabetes for 15 years, had a usual source of care, went to the doctor regularly, and reported an interest in her health. In fact, she asked the research nurse many questions about her diet and health during the interview. Yet she had been hospitalized for diabetes-related complications twice before in the previous two years.

To reduce PPH for Patient B, providers will need to better communicate the seriousness of diabetes and its management, test her knowledge comprehension through teach-backs, schedule more frequent outpatient visits, and perhaps engage family members in checking for symptoms.

#### *Example 3: Denial/*Avoidance

*“It [extreme heart failure symptoms] came on. I went to the doctor, hospital, [but]…. in a couple of weeks everything is back to normal, and I’m back at the Burger King and everything else. This time around it’s like it’s different. Those first two times around…I didn’t feel like I was close to death…I didn’t take my recovery too seriously.”*

Patient C is a 57-year-old male who has had congestive heart failure for over 10 years. Precipitating this admission, he received a shock from his pace-maker and thought he would die. He was also having headaches and indigestion. These were his *immediate* reasons for hospitalization. His *precipitating* reasons were that he was not following his diet and not exercising. The *underlying* reasons, in his description, were that he didn’t take his disease seriously. He says that after this hospitalization, he is determined to embrace recovery as he previously did sobriety 18 years earlier. *“This time I feel like I’m in trouble—that’s my motivation.”*

For Patient C, like with many individuals who describe denial/avoidance as their main pathway, the hospitalization itself was described as a wake-up call. Leveraging the opportunity and motivation of this moment may be very useful to reduce PPH.

#### Example 4: Behavioral Health and Health Care System (in combination)

*“I keep coming to the doctor and my health- not get any better.”*

Patient D is a 39-year-old female with diabetes covered by Medicaid and taking care of three children and her elderly mother. Her *immediate* reason for her hospitalization was MRSA, for which she has already been hospitalized during the past year. Her *precipitating* reason for hospitalization was not following her chronic care management plan. The *underlying* reasons included both behavioral health and health care system issues.

Patient D is an active abuser of methamphetamines who reports self-medicating to reduce pain because she could not get authorization for better pain pills due to a substance-use history. She says she doesn’t trust doctors and they really don’t trust her. She also feels that her doctor doesn’t communicate with her, that her specialists don’t communicate with each other, and that none of them communicate with the pharmacy. At one point, she notes, she was taking “*112 pills in a day*” and had 6 specialists. When probed if they were communicating, she states:

*“None were. When they would prescribe me medications, they wouldn’t even check in my file for if I already had such a thing or such a medication. So they were prescribing me all the pain killers, all these stuffs that were only going to make me feel sick. And I was wondering why when I’m taking all the medicines that they give me and my health not getting any better… But when I go research, a lot of the meds no mix with each other.”*

She felt that compliance with the non-coordinated medication plan actually caused her harm. Patient D would benefit from a relationship with her doctor that could help her reduce her substance abuse and could provide practical solutions for pain management and chronic care management in the meantime. Patient D noted that she appreciated the structure and dictated schedule of the hospital, and felt it would be helpful for her to have a similar schedule to follow at home.

#### *Example 5:* Practical problems

*“I don’t know why on that day I never eat anything…I had one party on my wife’s side…and I didn’t eat too much because I had two parties to go to.”*

Patient E is a 41-year-old male who has had diabetes since age 28. His *immediate* reason for hospitalization was hypoglycemia because he collapsed. His *precipitating* reason was that he took insulin with no food in his system. His primary *underlying* reason was practical as he described a busy weekend in which he did not eat much. Of the 90 people interviewed, only a few were like Patient E who described no other particular barriers in knowledge, access, behavioral, or social factors. Patient E described good social support and good general knowledge of how to care for his illness. Like many others coded for practical problems, Patient E appeared to be leaving the hospital with a new, very feasible plan to avoid a PPH. As he states: *“My plan is to try change…try to get my food ready before I take my insulin.”* Given his resources, it seems very likely that Patient E will not have another PPH.

## Discussion

In a predominately insured population, factors well outside the traditional purview of the hospital or even clinical medicine were critical underlying factors precipitating PPH. These findings supports the small, but growing line of research showing social factors to be related to PPH [4–11]. The few studies that have explored this issue with qualitative data have found patient experiences to be heterogeneous and not easily classified as “preventable” or “not preventable” [8]. Our research adds to this important literature with details about specific pathways that may help determine where within or outside the health care system this hospitalization might be preventable and direct interventions. We also provide new insights about PPH from a sample with substantial Asian American and Pacific Islander populations. This study is also relevant to current policy action around readmissions, as 66 % of individuals in this study had at least one readmission, with two as the average number of readmissions.

The three levels in the model correspond to how responsibility might be assigned for these hospitalizations. The *immediate* factor is often the focus of the clinical intervention. It is easy to blame the immediate causes—shortness of breath, infections—and try to focus clinical and technological solutions to resolve these issues. Sometimes we look beyond these to more “systems” issues, called *precipitating* factors in the model. We may find that a patient is hospitalized because of not following a chronic care management plan. Without looking further, providers may “blame the victim” and/or assume that better patient education may solve the problem.

But, as seen in many of our narratives, *underlying* factors may be the true drivers of the situation. For example several patients in this study with heart disease were hospitalized after excessive fluid intake, but they did so because they were homeless and/or had no air conditioning in a tropical climate. They knew that, according to their chronic care management plan, they should not drink water, but they were still hot. To reduce this behavior, it is important for a management plan to include practical considerations given these circumstances. This lack of attention to social and behavioral context may help explain the failure of many medically-focused, disease-management models in preventing hospitalizations [23].

Importantly, many of the factors that patients described as important, such as homelessness, limited patient knowledge, and poor health care system coordination/communication, are unlikely to appear in the hospital administrative data that have been the source of most analyses on this topic. Even factors such as mental illness and substance use, that could be measured in administrative data (albeit likely underreported), have not often been included in analyses of PPH. Additionally, multiple precipitating factors are seen in many cases [24]. Providers need to understand these background factors, which should be reflected in the medical record.

While hospital social workers and/or discharge planners may assess and document such relevant information, challenges remain. At many hospitals, social workers do not automatically see all patients. Also, patients may not feel comfortable admitting social concerns and/or existing questions in discharge planning may not fully capture social needs. For instance, instead of saying that they are homeless, patients may say that they are staying with family. This would satisfy discharge plan requirements to have somewhere safe to stay upon discharge, but would not illuminate the full scope of patients’ actual social needs. Even when providers know that patients have social challenges, this may be hard to find in the medical record as it may not be in the ‘Problem List’ or coded as a 'Condition.'

Patient perspectives**,** including a more complete story of their experiences**,** are vital to understanding how significant background issues create and/or exacerbate challenges in primary care and chronic care management. Without the patient narratives in this study, in many cases the *underlying* factors might not have been identified. If left with only the *immediate* and the *precipitating* factors to understand these hospitalizations, almost all the participants described in detail would look similarly non-compliant with medical advice. However, the pathways from the model seen in Fig. 2 reveal that they are non-complaint for very different reasons.

Recent efforts to better understand how to integrate social factors with administrative data can help expand our research and clinical knowledge around this topic [22] and may help direct interventions. Some patient-reported background factors were subtle, not readily measured with yes or no questions, including patients’ challenges related to trusting the doctor. Learning more about this, ideally in the outpatient setting, is an important to PCOR efforts to make interventions and outcomes specifically relevant to patients.

Our findings also question the notion of “potentially preventable hospitalization.” Clearly, some of the hospitalizations described here could not have been prevented solely by timely outpatient care [25]. Yet addressing social/contextual information in the clinical setting necessitates time and relationship-building, which are not easy to achieve given the functional and reimbursement limitations of our current health care system [26].

Our findings build on recent models of social context in potentially preventable readmissions. Our patient-reported factors mesh well with those reported by Cavillo-King et al. [10] from a systematic literature review as the “higher level social factors” less frequently studied, but critical to understanding readmissions. Similarly, Hersh et al. [27] provide a model of heart failure readmissions with social-contextual factors as critical. Our study confirms the value of such factors from the patients’ perspective as fundamental drivers to clinically significant outcomes.

Our findings are also relevant to the Leppin et al. [28] model of cumulative complexity (CuCoM). As they describe: “*Workload consists of all the work of being a patient and includes efforts to understand and plan for care, to enroll the support of others, and to access and use health care services. Capacity is determined by the quality and availability of resources that patients can mobilize to carry out this work”* ([27], p 1096). We provide specifics of social and behavioral factors that limit capacity and increase workload among vulnerable individuals, resulting in a potentially preventable readmission. These findings also provide insights for current work on risk models for PPH using administrative data [29]. Most current readmission risk prediction models perform poorly [30]. This study suggests that without further enrichment from social and behavioral factors, administrative data models will remain limited.

Current federal policies include penalties for hospitals with preventable readmissions [3]. Our study provides evidence that many of these hospitalizations may not be truly preventable within the traditional purview of the health care system. Innovative partnerships or policies should be developed to address these issues beyond the hospital, including linkages to social services and behavioral health [28, 31]. Policies, including sanctions for hospitals for preventable readmissions among vulnerable communities, should account for the fact that the precipitating factors for these hospitalizations (and many meaningful solutions to reduce them) lie beyond the hospital and demand a more integrated health care system and involvement of other domains both within the health care system (primary care) and outside it (housing, transportation services). These are particularly relevant in light of recent work confirming the importance of community factors in predicting hospital readmission rates [32]. This study helps to address concerns that CMS sanctions will unfairly impact safety net hospitals or that efforts to address PPH that do not consider patient context and vulnerability may lead to widening disparity gaps [33–36]. Thus, metrics measuring changes in PPH by hospital may be more appropriate than cross-sectional comparisons.

This study adds to literature by providing a model of pathways to PPH derived from patient perspectives. Compared to previous studies on this subject using qualitative data [8], we have a significantly larger sample. However, the study does have some limitations. We focused on HF and DM-related PPH. Different types of preventable readmissions (e.g., pneumonia) may be driven by other factors. Also, patients may not identify all factors influencing their health status. Other social, contextual, and health care-related factors beyond those studied here may also be important. For instance, community factors, including income inequality, have been associated with increased risk of readmission [7, 32], and factors such as “higher continuity of ambulatory care” [37] and “improving nurses’ work environments and staffing” have also shown promise to reduce preventable hospitalizations [5]. Future work should consider how these issues might intersect with patient-reported factors.

The AHRQ PPH metrics are designed for use with administrative discharge data [2] and have detailed exclusions (e.g., transfers from a skilled nursing facility), which we did not use as we accrued patients prior to discharge. Thus, we used the general disease categories to identify potentially eligible individuals, which may not correspond completely with the AHRQ PPH definition. Also, located in Hawai‘i, our study included a high proportion of Asians and Pacific Islanders. However, we believe the findings are relevant to other low-income, minority, and immigrant populations in the US.

A high proportion of patients with a PPH for CHF or DM at the hospital were not interviewed because of limited-English proficiency or mental health status, precluding informed consent. For these individuals, interventions may need to include caregivers. Interventions for those with limited-English proficiency may also need to be offered in-language, both in the hospital and in primary care. These variables are also unlikely to appear on administrative data and are not typically considered in analyses on this topic. This should be an area for future research. Also, all interviewers were female, which may have impacted patient disclosure. We used a $20 gift card as a study incentive. Following best practices, this amount was considered non-coercive; however, use of this incentive may have introduced bias in our sample, particularly as the incentive may have been more appealing to patients in greater economic need [38, 39].

Despite our focus on challenges, participants noted many positive aspects to their health care. For instance, while many participants mentioned challenges in communication with their health care providers, others mentioned strong, positive relationships. Because of the structure of this study, we could not measure the role of these strengths and weakness in predicting PPH, nor could we compare these individuals to those who were not hospitalized but have similar social and behavioral health challenges. Future work in an outpatient setting to see who is hospitalized and who is not hospitalized among those with similar circumstances would be useful and help answer questions arising from this work such as: What factors are protective? Which challenges matter the most?

## Conclusions

In a predominately insured population, factors well outside the traditional purview of the hospital or even clinical medicine were critical precipitating factors for many PPH. Patient perspectives were vital to understanding this issue. This information is unlikely to show up in administrative data and may not be discussed in the clinical encounter, but is critical in determining which PPH are truly preventable. Innovative partnerships/policies should be developed to address these issues, including linkages to social services and behavioral health. Policies including sanctions for hospitals for preventable readmissions, particularly for hospitals who serve vulnerable communities, should account for the fact that the precipitating factors for these hospitalizations (and many meaningful solutions to reduce them) lie beyond the hospital.

## Abbreviations

DM, diabetes; HF, heart failure; IRB, Institutional Review Board; PCOR, patient-centered outcomes research; PPH, potentially preventable hospitalization; QMC, Queen’s Medical Center

## Additional files

Additional file 1: Preventable Hospitalizations and Rehospitalizations Project, abridged questionnaire. (DOCX 45 kb) Additional file 2: Preventable Hospitalizations and Rehospitalizations Project, abridged items from the Electronic Medical Record Review form. (DOCX 49 kb) Additional file 3: Consolidated criteria for reporting qualitative studies (COREQ): 32-item checklist. (DOCX 27 kb)
