# Supplementary material for: Pathways to potentially preventable hospitalizations for diabetes and heart failure: a qualitative analysis of patient perspectives
Source: BMC Health Serv Res. 2016 Jul 26;16:300. doi: 10.1186/s12913-016-1511-6 (PMC4960879; doi:10.1186/s12913-016-1511-6)
Supplement: Additional file 1: — Preventable Hospitalizations and Rehospitalizations Project, abridged questionnaire. (DOCX 45 kb) [file 12913_2016_1511_MOESM1_ESM.docx]

Additional file 1. Preventable Hospitalizations and Rehospitalizations Project, abridged questionnaire

**Introduction**

Thank you for agreeing to participate in this study. We are about to begin an interview, which will take about 45 minutes to complete. Your answers are important to us. There are no right or wrong answers, just those that help us to understand your situation. You can always refuse to answer any question or group of questions without any impact on your care here at [THIS HOSPITAL]. Please let me know if you are in any discomfort or otherwise would like to stop the interview. We can always take a break and start again if that is easier for you.

Are you ready to begin?

YES 1

NO 2 (END interview)

**Give Rapid Estimate of Adult Literacy in Medicine** [3-5 minutes]

[If they mention that their eyesight is bad, ask if they would like a magnifying lens.]

NOTES: ________________________________________________________________________________________________________________________________________________________________________________

**Demographics**

Now I am going to ask you some questions about you and your family.

1. What is the highest grade or year of school completed?

(READ ONLY IF NECESSARY)

1 - Never attended school/only kindergarten

2 - Grades 1 thru 8 (Elementary)

3 - Grades 9 thru 11 (Some high school)

4 - Grades 12 or GED (high school grad)

5 - College 1 to 3 yrs (some college/Tech)

6 - Bachelor's Degree (BS, BA)

7 - Graduate degree (MA, MS, PHD)

8 - [DON'T KNOW]

9 - [REFUSED]

1. **Do you have health insurance?**

1 - No

2 – Yes

88 - [DON'T KNOW]

99 - [REFUSED]

1. What race do you consider yourself to be? (Can choose more than 1)

(READ LIST ONLY IF NECESSARY)

01 - White/Caucasian (European, German, Irish, Italian, English)

02 - Hawaiian

03 - Chinese (Taiwanese)

04 - Filipino

05 - Japanese (Okinawan)

06 - Korean

07 - Vietnamese

08 - Asian Indian

09 - Other Asian (Laotian, Thai, Malaysian) (SPECIFY)

10 - Samoan/Tongan

11 - Black/African American

12 - Native American/Aleut/Eskimo/Inuit

13 - Puerto Rican

14 - Mexican

15 - Portuguese

16 - Guamanian/Chamorro

17 - Other Pacific Islander (Polynesian, Micronesian, Fijian) (SPECIFY)

18 - Other (SPECIFY) _______________________________________________

88 - [DON'T KNOW/NOT SURE]

99 - [REFUSED]

1. **If more than one above, what would you describe as your primary race/ethnicity?**

Specify_______________________________________________

88 - [DON'T KNOW/NOT SURE]

99 - [REFUSED]

1. **Is there a place that you usually go to when you are sick or need advice about your health?**

1 - No

2 – Yes

88 - [DON'T KNOW]

99 - [REFUSED]

1. **Family income is important in analyzing the data we collect and is often used in scientific studies to compare groups of people who are similar. Please remember that all the data you provide is confidential.Of these income groups, which category best represents (your/the total combined family) income during 2012? (Remember, a family is a group of two or more people who live together and who are related by birth, marriage, or adoption.)**

Less than $4,999 …………………………………..1

$5,000-$9,999 2

$10,000-$19,999 3

$20,000-$29,999 4

$30,000-$39,999 5

$40,000-$49,999…………………. 6

$50,000-$74,999 ……………………………………7

$75,000-$99,999 8

$100,000-$199,999 9

$200,000 or more 10

DON’T KNOW 88

REFUSED…………………………………………99

For this next section, we are going to record your answers so we can remember everything you say. During this time, we will not call you by name to maintain your privacy in this recording.

*****Start Recording on Tablet Computer******

**Open-Ended Questions**

|  |
| --- |
| Give me a sense of what was going on at home and with your health before you came to the hospital. What convinced you to go to the hospital when you did? |
| Thinking over the past 2 weeks, were there any days when you did not take your medicine? If so, why? For example, some people don’t take their medicine because it makes them feel worse or because they already feel much better. |
| What do you think happened with your heart problem/ diabetes that you got sicker and had to come back to the hospital? |
| Is there anything different that could have been done to prevent you from coming to the hospital? Anything your doctor could have done? |
| When is the best time for you to learn about your heart /diabetes...do you remember much of what they said when you were discharged from the hospital? |
| What was the most important information or help you had when you were discharged from the hospital? |
| Are there any things you will do differently when you go home from the hospital this time? |

*****Turn off Recording on Tablet Computer******

I have turned off the recorder. That is all of the conversation that we will record from this interview.

**END**

Thank you. That completes all the questions that I have for you today.
