# Supplementary material for: Pathways to potentially preventable hospitalizations for diabetes and heart failure: a qualitative analysis of patient perspectives
Source: BMC Health Serv Res. 2016 Jul 26;16:300. doi: 10.1186/s12913-016-1511-6 (PMC4960879; doi:10.1186/s12913-016-1511-6)
Supplement: Additional file 2: — Preventable Hospitalizations and Rehospitalizations Project, abridged items from the Electronic Medical Record Review form. (DOCX 49 kb) [file 12913_2016_1511_MOESM2_ESM.docx]

**Additional file 2. Preventable Hospitalizations and Rehospitalizations Project, abridged items from the Electronic Medical Record Review form**

**Hospitalized For**

DM Short-Term Complications………………………………………… [ ]

DM Long -Term Complications ……………………………………….. [ ]

DM Lower Extremity Amputation…………………………………....... [ ]

Congestive Heart Failure………………………………………………. [ ]

1. **Demographics**
2. Age ___________
3. Gender ___________
4. **Cardiac History (If relevant)**
5. During the past 12 months, other than this stay, admitted to the hospital overnight or longer for CVD [ICD-9]?

NO 0

YES 1

DON’T KNOW 88

1. If yes, how many? _______
2. **DM History (If relevant)**
3. During the past 12 months, other than this stay, admitted to the hospital overnight or longer for diabetes [ICD-9]?

NO 0

YES 1

DON’T KNOW 88

1. If yes, how many? _______
