# Supplementary material for: Pathways to potentially preventable hospitalizations for diabetes and heart failure: a qualitative analysis of patient perspectives
Source: BMC Health Serv Res. 2016 Jul 26;16:300. doi: 10.1186/s12913-016-1511-6 (PMC4960879; doi:10.1186/s12913-016-1511-6)
Supplement: Additional file 3: — Consolidated criteria for reporting qualitative studies (COREQ): 32-item checklist. (DOCX 27 kb) [file 12913_2016_1511_MOESM3_ESM.docx]

**Additional file 3. Consolidated criteria for reporting qualitative studies (COREQ): 32-item checklist**

Sentell et al, submission

**Domain 1: Research team and reflexivity**

***Personal Characteristics***

1. Interviewer/facilitator: Which author/s conducted the interview or focus group?

MV, MY, and TS conducted the interviews. The majority were conducted by MY.

2. Credentials: What were the researcher’s credentials? E.g. PhD, MD

MV (RN), MY (RN), TS (PhD). The research team also included an MD, a DrPH, and a ScD.

3. Occupation: What was their occupation at the time of the study?

The interviewers’ occupations were: MV (Clinical Research Nurse), MY (Clinical Research Nurse), TS (Assistant Professor).

4. Gender: Was the researcher male or female?

All interviewers were female.

5. Experience and training: What experience or training did the researcher have?

All 3 interviewers had over 10 years and experience conducting research projects particularly focused on health disparities in populations of relevance to Hawaii, including qualitative and focus group interviewing. The research nurses have over 10 years of experience providing clinical care to a similar patient population as the participants included in this study and in conducting research at the focal hospital.

***Relationship with participants***

6. Relationship established: Was a relationship established prior to study commencement?

Participants met interviewers on the day of the interview. Rapport was built by asking semi-structured interview before open ended questions so the participant and the interviewer had time to get to know each other.

7. Participant knowledge of the interviewer: What did the participants know about the researcher? e.g. personal goals, reasons for doing the research

All participants received, discussed, and signed an IRB document that indicated the purpose of the study, including the following information.

***WHAT IS THIS PROJECT ABOUT?***

*We are doing this project to learn more about the people who have been admitted to the hospital for heart disease or diabetes problems. We want to learn more about you and your experience of how you manage your heart disease, diabetes and overall health when you are at home (and not in the hospital). We also want to find out about any difficulties or concerns you have about managing your heart disease or diabetes at home, and what happened when you got sick and you had to come to the hospital. This information we collect will help us to better understand how people with heart disease and diabetes manage their care at home and also why they sometimes need to come back to the hospital for care of their heart or diabetes problems.*

Participants did not know personal information about the interviewer besides their name and the fact that they were conducting this study.

8. Interviewer characteristics: What characteristics were reported about the interviewer/facilitator? e.g. Bias, assumptions, reasons and interests in the research topic.

The fact that all interviewers were female is included in the limitations section of the manuscript. While many participants were male, gender was not a particular component of the research question and we do not think this introduced a major bias.

**Domain 2: study design**

***Theoretical framework***

9. Methodological orientation and Theory: What methodological orientation was stated to underpin the study? e.g. grounded theory, discourse analysis, ethnography, phenomenology, content analysis

The methodological orientation used was the framework analysis approach, which is a specific method related to content analysis.^1,2^ This approach was chosen as having particular utility for this research project for the following two reasons.

**First**, this approach allow for very specific expectations about some of the findings you will have while also allowing room for emerging themes that may arise. In this particular case, we went into the research project familiar with relevant research, including the large, existing literatures on (1) social factors related access to health care, (2) patient challenges in understanding health information, and (3) medication adherence. We were, in fact, inspired to do this project because these large existing literatures did not appear to be well integrated with the policy-relevant issue of potentially preventable hospitalization (PPH), which have been largely conducted with administrative data in which patient’s social, knowledge, and medication adherence challenges are not typically recorded in detail. We also used the behavioral model of health care utilization as a conceptual model,^3^ which proposes an array of predisposing, precipitating, and need characteristics to be relevant to health care use and outcomes. Again, only some of these characteristics are typically captured in administrative data typically used to study determinants of PPH. We wanted to hear patient stories about which of these factors, including those we could not obtain with quantitative analyses of administrative data were relevant to them.

At the same time, we did not know what patients who were hospitalized with these particular “preventable conditions” would report and in what combinations. Would their challenges be all access to care, confusion with drugs, lack of knowledge about complex chronic care management plans, unknown factors, or combinations of these? Thus, the framework approach gave us a method that would allow us to acknowledge that we already had a good sense of many factors related to poor health outcomes generally and PPH specifically, but that we expected new factors and specific combinations of these factors to emerge from patient stories.

**Second,** the framework approach specifically considers patterns both across cases and the nexus of those factors within cases. We were interested not just in the factors participants mentioned as important to their hospitalizations, but also what those patterns looked like within individuals. A study goal was to not only identify any unexpected new factors, but also to consider the array of factors for specific patients and their relevance to PPH and intervention opportunities. Thus, we went in knowing we would find an array of factors that would be related to PPH, but only through patient stories were we able to understand the specific levels of pathways that are demonstrated in our final model and how those manifest both across (general model) and within specific patients (general model used in examples).

***Participant selection***

10. Sampling: How were participants selected? e.g. purposive, convenience, consecutive, snowball

Potentially eligible subjects were identified by one or two ways.  First, the patients’ attending physician could identify and refer potentially eligible subjects to the research team, who then approached the patient to obtain informed consent.  Attending physicians at The Queen’s Medical Center for these patients were cardiologists (for heart failure), hospitalists or other internists (for DM complications and heart failure), or vascular surgeons (for lower-extremity amputations). Second, advance practice nurses (APRNs) who were part of the care team also identified potentially eligible subjects, and notified the research team. During the recruitment period, the research nurse checked in with the relevant providers approximately two times a week to discuss the study and identify patients who met inclusion criteria.

Eligible subjects include patients with a primary admission diagnosis of at least one of the following 4 conditions, as defined by the Agency of Healthcare Research and Quality (AHRQ): heart failure, uncontrolled diabetes, short-term (e.g., ketoacidosis, hyperosmolarity) and long-term (e.g., renal, eye, circulatory) diabetes complications, and lower-extremity diabetes-related amputations. Because the research was Hawai‘i-based, focal racial/ethnic groups were Asians, Native Hawaiians, other Pacific Islanders (e.g., Samoans, Tongans, Micronesians), and whites. Participants had to be comfortable speaking English.

Eligible subjects were identified initially by the clinician. From subjects flagged as potentially eligible by clinicians, a trained Nurse Researcher then obtained permission from the attending physician and approached the patient to see if they were willing to take part in the study including a 40-minute interview and the electronic medical record. At that point, the Nurse Researcher screened for full study eligibility.

Additional eligibility criteria include: 1) Age >21 year old and 2) Willing to participate in semi-structured interview and answer survey questions.

Exclusion criteria include: 1) Any patient in ICU; 2) Clinically unstable; 3) Non-English speaker; 4) Did not self-identify as Asian, Native Hawaiian, other Pacific Islander, or white, 5) Pregnancy; 6) Memory loss or inability to participate in interview; 7) Non-Hawaii resident; 8) Resident of nursing home, hospice, prison, or other similar institution.

11. Method of approach: How were participants approached? e.g. face-to-face, telephone, mail, email

Face to face.

12. Sample size: How many participants were in the study?

90

13. Non-participation: How many people refused to participate or dropped out? Reasons?

No one dropped out as this was a cross-sectional study. A considerable portion of individuals identified as potentially eligible by providers (n=393) were ineligible for the study interview (n=238). Top reasons were altered mental status, including dementia (n=82), limited English proficiency (n=76), and being too ill to participate (n=34). Among those otherwise eligible, 30 refused participation, mostly stating they were not interested in the topic. An additional two individuals were deemed ineligible for study inclusion after interview completion; one admission was for a congenital heart issue, not a preventable condition, and one was for chronic obstructive pulmonary disease, not a study focus.

***Setting***

14. Setting of data collection: Where was the data collected? e.g. home, clinic, workplace

All data was collected at the hospital.

15. Presence of non-participants: Was anyone else present besides the participants and researchers?

In some cases, patient’s friends, family members, or another patient were in the room. These individuals were not interviewed.

16. Description of sample: What are the important characteristics of the sample? e.g. demographic data, date

This is described in Table 1 of the manuscript.

***Data collection***

17. Interview guide: Were questions, prompts, guides provided by the authors? Was it pilot tested?

The open-ended interview questions were extensively pilot tested before implementation and were also evaluated and revised based on guidance from other experienced interviewers. Open-ended questions are now included as a study appendix. As patients were telling their stories, open ended questions were supplemented with prompts about areas of relevance and interest to ensure all relevant detail was covered by the experienced patient interviewers.

18. Repeat interviews: Were repeat interviews carried out? If yes, how many?

Most patients were only interviewed once. One patient with several readmissions was interviewed two more times following each readmission. She is included only once in this study.

19. Audio/visual recording: Did the research use audio or visual recording to collect the data?

Interviews were recorded using an ipad.

20. Field notes: Were field notes made during and/or after the interview or focus group?

Field notes were taken during the interview and also immediately after the interview. These were considered in analyses.

21. Duration: What was the duration of the interviews or focus group?

Interviews took approximately 40 minutes.

22. Data saturation: Was data saturation discussed?

Data saturation was discussed and the study was closed following thematic saturation.

23. Transcripts returned: Were transcripts returned to participants for comment and/

or correction?

Transcripts were not returned to participants for comment and/or correction. Our IRB study protocols did not include patient follow-up.

**Domain 3: analysis and findings**

***Data analysis***

24. Number of data coders: How many data coders coded the data?

Two independent coders (TS and MQ) coded the data.

25. Description of the coding tree: Did authors provide a description of the coding tree?

The framework approach uses a coding matrix. We provide descriptions of our coding process in the manuscript.

26. Derivation of themes: Were themes identified in advance or derived from the data?

Both as described above from the framework analyses.

27. Software: What software, if applicable, was used to manage the data?

Excel and word were used to manage the thematic data.

28. Participant checking: Did participants provide feedback on the findings?

Our IRB study protocols did not include patient follow-up. (We have conducted three stakeholder focus groups following presentations of study findings to obtain community thoughts about their relevance and correctness. Responses have indicated that our findings mesh well with community experiences.)

***Reporting***

29. Quotations presented: Were participant quotations presented to illustrate the themes/findings? Was each quotation identified? e.g. participant number

Yes.

30. Data and findings consistent: Was there consistency between the data presented and the findings?

Yes.

31. Clarity of major themes: Were major themes clearly presented in the findings?

We hope so. This is the goal of the overall model in the paper.

32. Clarity of minor themes: Is there a description of diverse cases or discussion of minor themes?

This is the goal of the examples in the paper.

**References**

1.Smith, J., Firth, J. (2011) Qualitative data analysis: application of the framework approach. Nurse Researcher, 18: 52-62

2. Gale NK, Heath G, CameronE, Rashid S, Redwood S. (2013). Using the framework method for the analysis of qualitative data in multi-disciplinary health research. BMC Med Res Methodol, 13: 117.

3. Andersen RM, Newman, JF. (1973).Societal and Individual Determinants of Medical Care Utilization in the United States. Milbank Memorial Fund Quarterly Journal, 51:95-124.
